# Supplementary material for: The splicing regulators Esrp1 and Esrp2 direct an epithelial splicing program essential for mammalian development
Source: eLife. 2015 Sep 15;4:e08954. doi: 10.7554/eLife.08954 (PMC4566030; doi:10.7554/eLife.08954)
Supplement: Figure 4—source data 2. — DOI: http://dx.doi.org/10.7554/eLife.08954.013 [file elife08954s002.docx]

| Supplementary file 2: RNAseq and RT-PCR validated SE Splicing events | | | | | | | |
| --- | --- | --- | --- | --- | --- | --- | --- |
| Gene Symbol | **Mm10**  **Chromosome location** | **RNAseq**  **WT PSI** | **RNAseq**  **DKO PSI** | **ΔPSI**  **(DKO-WT)** | **RT-PCR**  **WT PSI** | **RT-PCR**  **DKO PSI** | **ΔPSI**  **(DKO-WT)** |
| Esrp Enhanced SE Events | | | | | | | |
| Map3k7 | chr4(+): 31994873-31994954 | 72.10 | 34.45 | -37.65 | 69.29 | 52.29 | -16.99 |
| Ralgps2 | chr1(-): 156821386-156821464 | 76.70 | 10.40 | -66.30 | 58.43 | 4.32 | -54.11 |
| Lef1 | chr3(+): 131191022-131191106 | 93.80 | 67.25 | -26.55 | 96.47 | 80.94 | -15.53 |
| Mllt4 | chr17(+): 13848354-13848375 | 97.00 | 71.25 | -25.75 | 99.43 | 88.92 | -10.51 |
| Enah | chr1(-): 181,911,597-181,911,659 | N/D | N/D | N/D | 53.98 | 0.07 | -53.91 |
| Numb | chr12(-): 83797196-83797343 | N/D | N/D | N/D | 35.97 | 5.86 | -30.11 |
| Nf2 | chr11(-): 4780577-4780622 | 92.50 | 9.35 | -83.15 | 95.24 | 11.33 | -83.91 |
| Epb4.1 | chr4(-): 131937014-131937464 | 93.25 | 9.50 | -83.75 | 59.50 | 9.02 | -50.48 |
| Myo6 | chr9(+): 80303272-80303299 | 91.50 | 4.95 | -86.55 | 93.35 | 3.06 | -90.29 |
| Fnbp1 | chr2(-): 31044881-31044896 | 58.30 | 2.65 | -55.65 | 39.26 | 1.10 | -38.16 |
| Ptprf | chr4(-): 118227818-118227845 | 70.40 | 0.00 | -70.40 | 82.35 | 0.01 | -82.34 |
| Macf1 | chr4(-): 123364056-123364074 | 86.25 | 7.10 | -79.15 | 79.38 | 3.53 | -75.85 |
| Itga6 | chr2(+): 71853533-71853663 | 95.10 | 56.10 | -39.00 | 95.42 | 56.07 | -39.35 |
| Rap1gap | chr4(+): 137723745-137723823 | N/D | N/D | N/D | 17.96 | 1.13 | -16.83 |
| Atp6v1c2 | chr12(-): 17288995-17289133 | 99.40 | 3.55 | -95.85 | 98.51 | 1.26 | -97.25 |
| Uap1 | chr1(-): 170147995-170148046 | 80.85 | 20.90 | -59.95 | 77.22 | 19.75 | -57.47 |
| Arhgef10l | chr4(-): 140568755-140568770 | 98.20 | 5.50 | -92.70 | 99.99 | 6.26 | -93.73 |
| Grhl1 | chr12(+): 24582884-24582961 | 100.00 | 19.50 | -80.50 | 99.04 | 13.64 | -85.40 |
| Esrp Silenced SE Events | | | | | | | |
| Fam213b | chr4(-): 154897348-154897424 | 74.90 | 99.65 | 24.75 | 66.07 | 99.99 | 33.92 |
| Tor2a | chr2(+): 32759526-32759702 | 73.65 | 98.70 | 25.05 | 70.98 | 99.99 | 29.01 |
| Timm17b | chrX(+): 7900960-7901060 | 52.00 | 99.45 | 47.45 | 28.53 | 99.80 | 71.27 |
| Arhgef11 | chr3(+): 87734423-87734552 | 7.85 | 74.40 | 66.55 | 0.70 | 54.73 | 54.04 |
| Scrib | chr15(-): 76061738-76061801 | 17.10 | 94.80 | 77.70 | 17.95 | 96.36 | 78.41 |
| Plekha1 | chr7(+): 130909587-130909628 | 13.25 | 82.50 | 69.25 | 2.68 | 68.94 | 66.26 |
| Lsm14b | chr2 (+): 180031793-180031871 | 14.25 | 97.75 | 83.50 | 11.70 | 97.69 | 85.99 |
| Arhgap17 | chr7(-):123294471-123294705 | 11.40 | 68.40 | 57.00 | 1.22 | 30.87 | 29.65 |
| Akap9 | chr5(+): 4004802-4004943 | 16.60 | 59.85 | 43.25 | 5.42 | 34.52 | 29.10 |
| Myo1b | chr1(-): 51766821-51766908 | 17.15 | 85.45 | 68.30 | 4.62 | 86.59 | 81.97 |
| N/D: Not detected by Epidermis RNAseq | | | | | | | |
